# Comparative Analysis of Root Canal Filling Debris and Smear Layer Removal Efficacy Using Various Root Canal Activation Systems during Endodontic Retreatment

**DOI:** 10.3390/medicina56110615

**Published:** 2020-11-16

**Authors:** Seong Yeon Park, Mo Kwan Kang, Hae Won Choi, Won-Jun Shon

**Affiliations:** 1 Department of Conservative Dentistry, College of Dentistry, Seoul National University, Seoul 08826, Korea; alpharigel@hanmail.net; 2 Department of Endodontics, University of California, Los Angeles, CA 90095, USA; mkkang@dentistry.ucla.edu; 3 Department of Dental Biomaterials Science, School of Dentistry, Seoul National University, Seoul 08826, Korea; orthochoi7@gmail.com; 4 Department of Orthodontics, Institute of Oral Health Science, Samsung Medical Center, Sungkyunkwan University School of Medicine, Seoul 06351, Korea

**Keywords:** retreatment, irrigation, ultrasonic, sonic, multisonic, scanning electron microscopic (SEM)

## Abstract

Background and objectives: The complete removal of obturation material can be a challenge in nonsurgical root canal retreatment. The insufficient removal of obturation material is a reason for root canal retreatment failure. Materials and Methods: The purpose of this study was to assess the efficacy of different final root canal irrigation activation methods in removing debris and smear layers in the apical and middle portions of root canals during retreatment. Sixty-six distal roots of freshly extracted molars were randomly divided into six groups: (1) primary root canal treatment with no obturation (negative control); (2) retreatment with only conventional instrumentation and irrigation (positive control); (3) retreatment with additional ultrasonic irrigation using the Piezon Master 700; (4) ultrasonic irrigation with the ENDOSONIC Blue; (5) sonic irrigation with the EDDY; and (6) multisonic irrigation with the GentleWave system. Roots were split and prepared for scanning electron microscopic (SEM) evaluation. Acquired images were assessed to quantify the amount of debris and smear remaining. Results: Among the treatment groups, Group 6 had a significantly lower debris score than Group 2 (positive control) in both the middle and apical regions (*p* = 0.004, *p* = 0.012). All treatment groups showed significantly lower smear scores than Group 2 in the middle and apical regions (*p* < 0.05). Conclusions: The GentleWave multisonic System showed a more optimal cleaning efficacy of the root canal debris but did not differ significantly with the tested passive ultrasonic or sonic irrigation method.

## 1. Introduction

In a survey conducted by the American Association of Endodontics, 46% of all endodontic treatments were nonsurgical retreatments [1]. The objective of a nonsurgical root canal retreatment is to restore healthy periapical and periradicular tissue [2]. Healthy periapical tissue can be restored only when the root canal system is free of any organic tissue, bacteria/biofilm, and inorganic dentinal debris and obturation material. However, removing the previously filled obturation material from a root canal system after a primary treatment remains a challenge [3,4].

The removal of gutta-percha using hand instruments is a slow and difficult process, especially when the filling material is well compacted. Thus, nickel–titanium (Ni–Ti) rotary instruments are recommended for reducing clinical time and facilitating removal. However, many studies showed that even after using Ni–Ti rotary instruments specifically developed for retreatment, filling material was not completely removed from the root canal walls [5,6,7,8]. The ability of ultrasonic irrigation to reduce the amount of remaining filling material following root canal retreatment was tested, and the results were compared to those of conventional needle irrigation and instrumentation [9]. However, in that study, none of the roots were free of gutta-percha and sealer.

Recently, several new irrigation systems and techniques have been developed. ENDOSONIC Blue (MARUCHI, South Korea) is a passive ultrasonic irrigation (PUI) system that consists of a Ni–Ti file without superelasticity at temperatures below 55 °C. At the temperature of NaOCl used for root canal treatment, the file has no superelasticity and is easily bent. Therefore, loss of ultrasonic energy could be reduced even if the file is in the curved root canal wall, and ultrasonic energy can be transmitted well into the apical region.

A novel passive sonic irrigation (PSI) system, EDDY (VDW, Munich, Germany), is powered to a high frequency up to 6000 Hz by an air scaler. The vibration produced is transferred to the polyamide tip, which is moved in an oscillating motion at high amplitude. This three-dimensional movement triggers cavitation and acoustic streaming.

Another endodontic device, the GentleWave System (Sonendo, Laguna Hills, CA, USA), delivers treatment fluids with minimal instrumentation to root canals using a combination of acoustics and advanced fluid mechanics [10]. A high-speed degassed treatment fluid is delivered into the pulp chamber of the tooth by a treatment instrument on the occlusal surface of an accessed tooth. The treatment fluid reaches the entire root canal system while built-in suction within the treatment instrument removes the excess fluid [11,12]. The GentleWave System has been shown to greatly remove tissue debris and biofilm from complex anatomical areas such as the isthmi [13,14].

The purpose of this study was the in vitro comparison of the cleaning efficacy of the GentleWave System and passive ultrasonic or sonic system during the retreatment of root canal systems that were conventionally treated and obturated with gutta-percha with AH Plus (Dentsply Rinn, Elgin, IL, USA). 

## 2. Materials and Methods

This study was approved by the Seoul National University human research ethics committee (IRB No. S-D20160011, Date 22 March 2016). We collected 66 freshly extracted human mandibular molars that were visually and radiographically examined and stored in phosphate-buffered solution at 4 °C. For study purposes, any teeth with large decay or fractures, internal or external root resorption, open apices, calcification of the root canals, or previous root canal therapy were excluded. The average distal root length was 12.3 ± 1.67 mm, and the average angle of curvature was 14.3° ± 7.7° for all mandibular molars.

When present, caries were removed. Missing coronal tooth structure was restored using etchant (Etch-Rite, Pulpdent, Watertown, MA, USA), bonding agent (Optibond, Kerr, Orange, CA, USA), and Virtuoso flowable light-cure composite (Denmat, Lompoc, CA, USA). Following endodontic access, all teeth were firmly secured using an adhesive (McMaster-Carr, Los Angeles, CA, USA) and sealed within a water-saturated porous medium to simulate blood-saturated periapical tissue. After endodontic access preparation, reproducible glide paths and working lengths (WLs) were established using #10 K files (MANI K-files, Utsunomiya, Japan). WL was defined as 1 mm from the radiographic apex.

### 2.1. Primary Treatments

Primary root canal treatments involved conventional rotary instrumentation and irrigation. Conventional rotary instrumentation used shaping files (SX, S1, S2) and finishing files (F1 and F2 files) (ProTaper Files, Denstsply, Tulsa Dental Specialties, Tulsa, OK) spun at a speed of 300 rpm [15]. During instrumentation, 2 mL 3% NaOCl (diluted with distilled water; Clorox, Oakland, CA, USA) was delivered between the use of each instrument. RC Prep (Patterson Dental, Saint Paul, MN, USA) was used as necessary. Final conventional irrigation was performed with 3% NaOCl solution using a 30G Max-i-Probe irrigation needle (Dentsply Rinn, Elgin, IL, USA) and a syringe for 1 min at a flow rate of 5 mL/min followed by a 2 min rinse with 17% EDTA (Pulpdent Corporation, Watertown, MA, USA) at 5 mL/min and a final rinse with 1 mL saline solution. During irrigation, the irrigation needle was placed 1 mm short of the WL and was moved with a 1–2 mm up-and-down motion to prevent locking in the canal. Canals were dried with sterile paper points (Dentsply). Eleven samples were randomly selected as negative controls (Group 1).

### 2.2. Obturation Following Primary Endodontic Treatments

Canals were obturated using a lateral condensation technique with gutta-percha cones (Dentsply) corresponding to the final file size and accessory cones (Diadent, Burnaby, BC, Canada) along with AH Plus sealer (Dentsply). Radiographs were acquired after obturation. Cotton plugs were placed in pulp chambers, and the access opening was covered with Cavit (3M ESPE, Seefeld, Germany). Teeth roots were wrapped in sterile, moistened cotton, placed in a vial labeled with the specimen number, and stored in an incubator at 37 °C for two weeks. Teeth were maintained in a moist environment throughout incubation.

### 2.3. Retreatment—Removal of Obturation Material

Cavit and cotton plugs were removed from pulp chambers and replaced with 100 µL chloroform (Patterson Dental) to soften the gutta-percha. ProTaper Universal Retreatment files D1, D2, and D3 (Dentsply) at a 500–700 rpm were used to remove gutta-percha [15]. Between instrument changes and during the removal of gutta-percha, canals were irrigated with 2 mL 3% NaOCl. When necessary, hand files were used to confirm patency.

After removing obturation material, canals were shaped further with conventional rotary instrumentation comprised of the ProTaper shaping files (SX, S1, and S2) and finishing files (F1, F2, and F3 files) at a speed of 300 rpm. Canals were irrigated with 2 mL 3% NaOCl between the use of each instrument. Radiographs were acquired after the removal of obturation material. Eleven samples were randomly selected as positive controls and used to establish baseline values for debris (Group 2). The other samples underwent additional irrigation to form Groups 3, 4, 5, and 6.

#### 2.3.1. Group 3: Passive Ultrasonic Irrigation System

A Piezon Master 700 with an ESI tip (size 15, taper 0.02) (DT-011, EMS, Nyon, Switzerland) was set to low-power Endo Mode with a medium-to-high irrigation flow rate, as recommended by the manufacturer. One reservoir of the Piezon Master 700 was filled with 3% NaOCl and another with 8% EDTA. The Piezon Master 700 with ESI tip was used to irrigate 3 mL/min for 10 s per canal and activate each canal for 3 × 20 s with 3% NaOCl. Similar activation was performed for 3 × 20 s with 8% EDTA. The 8% EDTA solution was selected based on recommendations in the instructions for the GentleWave System. Each canal was irrigated with 2 mL distilled water. All canals were dried with sterile paper points.

#### 2.3.2. Group 4: Passive Ultrasonic Irrigation System

An ENDOSONIC Blue ultrasonic system with a Ni–Ti file (size 17, taper 0.02) was set to maximum power, and 3% NaOCl was delivered to each canal by syringe needles per 10 s. The activation of each canal with the ENDOSONIC Blue was for 5 × 10 s, as recommended by the manufacturer. Root canals were flushed with 3% NaOCl. Similar activation was performed for 5 × 10 s with 8% EDTA. Each canal was irrigated with 2 mL distilled water. All canals were dried with sterile paper points.

#### 2.3.3. Group 5: Passive Sonic Irrigation System

EDDY was set to power 2, as recommended by the manufacturer. EDDY was activated in each canal for 3 × 20 s with 3% NaOCl and 3 × 20 s with 8% EDTA. Each canal was irrigated with 2 mL distilled water. All canals were dried with sterile paper points.

#### 2.3.4. Group 6: GentleWave System

For the GentleWave treatments, the tip of the treatment instrument was placed inside the pulp chamber of the molar. The treatment consisted of 3% NaOCl for 5 min, distilled water for 30 s, 8% EDTA for 2 min, and distilled water for 15 s as recommended by the manufacturer [13]. All canals were dried with sterile paper points.

### 2.4. Post Processing

**Figure 1:**
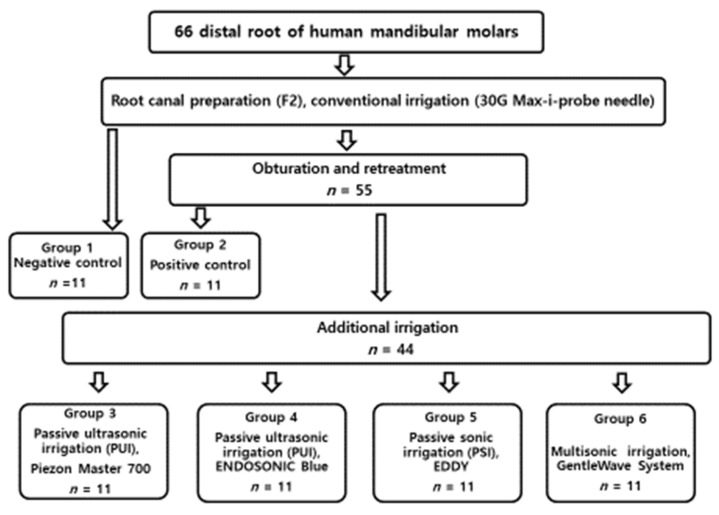
Flowchart of the all the experimental procedure.

Each tooth was filled with 1 mL 4% buffered formalin for overnight fixation at 4 °C. The roots from the six groups were separated from their crowns with a diamond disc (NTI, Rotary Dental Instruments, Kahla, Germany). The roots were split longitudinally, and representative specimens were sectioned horizontally at 5 mm from the anatomic apex. Samples underwent standard dehydration processing with ascending grades of ethanol (50, 70, 80, and 100%) for 10 min with further dehydration in 100% ethanol for 30 min. Samples were mounted on tabs with carbon conductive tape and sputter coated with Au–Pd at 20 mA for 1 min. All the treatment flows are illustrated in Figure 1.

### 2.5. Scanning Electron Microscopy (SEM) Imaging and Scoring

For each root half, SEM images were acquired in the apical (1–3 mm) and middle (4–6 mm) regions. Images were acquired at 40×, 150×, and 600× at 15 kV using a SEM (Hitachi TableTop TM3010, Krefeld, Germany). For the analysis, debris was defined as the residual filling material and dentinal mud in the canal area. A modified scoring method was used to assess the amount of debris and smear [16]:

For the debris score:

0: Clean canal wall, very few debris particles; <10%;
1: Few small residual debris; 10–25%;
2: Many residual debris, <50% of the canal wall covered; 25–50%;
3: >50% of the canal wall covered; 50–75%;
4: Complete or nearly complete covering of the canal wall by debris; 75–100%.

For the smear score:

0: No smear layer, orifice of dentinal tubules patent; 90% or more open dentinal tubules;
1: Small amount of smear layer, some open dentinal tubules; 50–90% open dentinal tubules
2: Homogeneous smear layer along most of the canal wall, few open dentinal tubules; 25–50% open dentinal tubules;
3: Entire root canal wall covered with a homogeneous smear layer, very few open dentinal tubules; <25% open dentinal tubules;
4: Thick homogenous smear layer covering the entire root canal wall. 0% open dentinal tubules.

Debris scoring was performed at ×150 magnification and smear scoring at ×600.

### 2.6. Data Analysis

Images acquired in the apical and middle regions for each root half were blindly evaluated by two calibrated examiners and averaged. Average debris and smear scores were calculated for each root. Differences between groups were compared using the Kruskal–Wallis test followed by a Tamhane T2 test at *p* < 0.05.

## 3. Results

**Figure 2:**
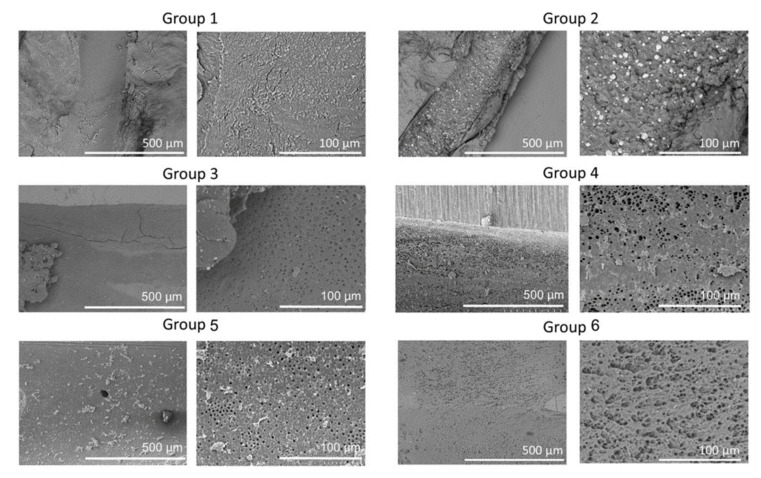
Representative scanning electron microscopy (SEM) images of the apical regions of root canals from the six groups. SEM images are the low (×150) and high (×600) magnifications of root canals showing clean, open tubules in Group 6 compared to other groups.

Representative SEM images of the six groups at 150× and 600× are shown in Figure 2. Group 1 showed less debris but very high smear. Group 2 showed large amounts of debris and smear; Groups 3, 4, and 5 showed less debris and smear; and Group 6 showed the smallest amounts of debris and smear.

**Figure 3:**
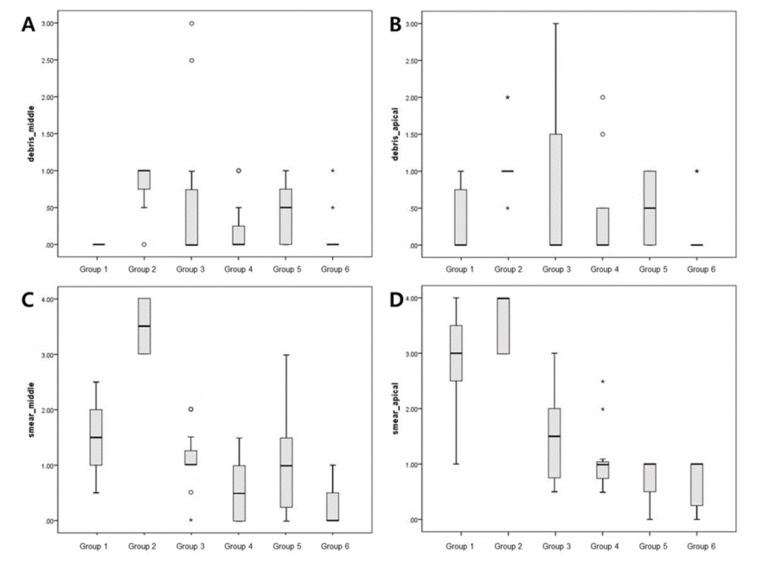
Box plots showing the distribution of values for the groups: (**A**,**B**) show the debris scores for the middle and apical regions, respectively; (**C**,**D**) show smear scores for the middle and apical regions, respectively. Small circles, mild outliers; asterisks, extreme outliers (individual values more than 1.5 interquartile range).

The evaluation of each root half provided similar semi-quantitative results (Figure 3, Table 1). According to the debris score analysis, Group 1 demonstrated the lowest debris score in the middle regions. Group 2 had the highest overall average debris score in both the apical and middle regions. Group 6 had the lowest debris score in the apical regions. Among the treatment groups, only Group 6 had a significantly lower debris score than Group 2 (positive control) in both the middle (*p* = 0.004) and apical regions (*p* = 0.012). Following an analysis of smear score, the highest smear score was for Group 2 in the middle and apical regions. Group 6 had the least amount of smear in the middle and apical regions. In the middle regions, Group 6 had significantly less smear than the control group (*p* < 0.001). In the apical regions, Groups 3, 4, 5, and 6 had significantly less smear than the control group (*p* = 0.005). Group 6 had the lowest debris and smear scores in the apical regions.

Table 1 provides the summary of the average debris and smear scores for apical and middle regions for the six groups.

**Figure 4:**
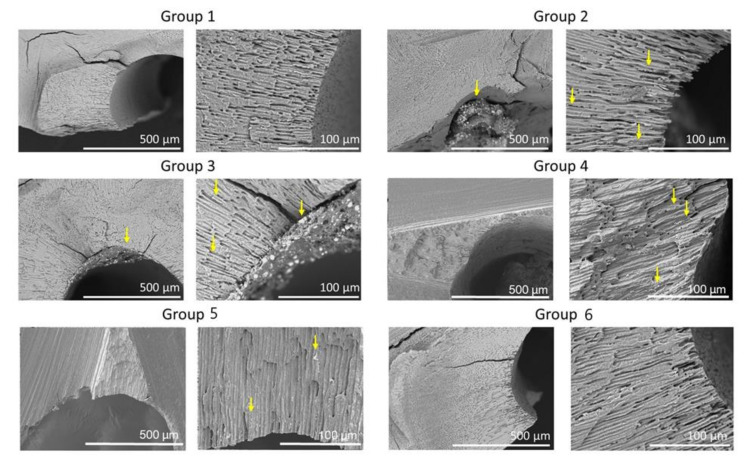
Representative SEM of the cross-sections of the six groups. SEM images show low (×150) and high (×600) magnifications of root canals. Note sealer remaining in dentinal tubules (arrows).

Figure 4 shows representative cross-sectional SEM images for the six groups. Cross-sectional images for Group 1 were similar to that of Group 6. SEM images of Groups 2, 3, 4, and 5 showed the presence of residual filling material in the canals and tubules.

## 4. Discussion

This study showed that the amount of debris removed in the apical and middle regions of root canal systems was greater with the GentleWave System than with ultrasonic or sonic activation. However, the differences between removal with GentleWave System and other devices were not significant. The positive controls, for which additional activated irrigation was not performed, showed similar debris scores to PUI/PSI groups. These findings agree with a study by Da Rosa, in which the greatest reduction in filling material was observed after using ProTaper Universal Retreatment files (*p* < 0.05), and PUI did not improve the removal of filling material after using rotary files for root canal preparation (*p* > 0.05) [17]. The lack of improvement in the debris score after using a passive ultrasonic or sonic irrigation method was probably because of the high bond strength of AH Plus to root dentin [18], and its adhesion was likely higher than the forces generated by acoustic microstreaming and cavitation. Overall, relatively clean canal walls were found among all groups. This result may be attributed to the root canal preparation size (F3), which allowed sufficient debris transportation coronally.

All treatment groups showed significantly lower smear scores than the control group in the apical regions (*p* = 0.005). In the middle regions, only the GentleWave System showed a significantly lower smear score than the control group (*p* < 0.001). The results in this study indicate that the use of activated irrigation is beneficial for removing smear layers in apical regions. These results are similar to those reported by Urban et al. [19] and Haupt et al. [20] who demonstrated a similar effectiveness regarding smear layer removal for PUI and PSI, and both activation techniques performed significantly better than syringe irrigation. Nevertheless, further studies are required to assess root canal cleanliness after canal preparation and the subsequent activation of irrigants with flexible tips in curved canals, as the influence of sonic and ultrasonic activation in severely curved root canals is controversial [20,21,22].

Sonic irrigation differs from ultrasonic irrigation because it operates at a lower frequency. For sonic application, frequencies range from 1000 to 6000 Hz. Consequently, the streaming velocity of the irrigant is lower. Moreover, the oscillating patterns of the sonic instruments are different, with one node near the attachment of the file and one antinode at the tip of the file. When the movement of the sonic file is constrained, the sideways movement disappears, while longitudinal vibration is produced [23]. Two studies report that PUI removes more dentine debris from root canals than sonic irrigation [24,25], and the positive relationship between streaming velocity and frequency is thought to explain the higher efficiency of PUI versus sonic irrigation. However, recent studies showed that EDDY tips activated by an air scaler at 6000 Hz performed equal to or better than PUI [19,20], in agreement with our study. Although EDDY generates lower frequencies than ultrasonic devices (25–30 kHz), it was equally effective in cleaning debris and smear layers. This result might be because the effect of cavitation is dependent on the frequency of the instrument inside the root canal and on the amplitude of the swinging instrument. Thus, cavitation might occur at lower frequencies [26]. Measurements show an amplitude of 11 µm for an ultrasonic instrument and 346 µm ± 41 µm for EDDY [26].

A significant difference was seen for debris scores between the GentleWave System and positive controls, as observed in SEM images. The GentleWave System disperses different treatment fluids from the tip of the handpiece into the pulp chamber. When the treatment fluid is in contact with stagnant fluids in the pulp chamber, because of shear forces, hydrodynamic cavitation occurs, forming thousands of microbubbles called a cavitation cloud. The bubbles subsequently implode and create sound waves that cover a broad frequency spectrum (multisonic ultracleaning spectra) that reverberate and contribute to the cleaning throughout the root canal system. The presence of multisonic ultracleaning energy in combination with advanced fluid dynamics loosens the obturation material and sealer when engaged in the dentinal wall and leads to a low debris score. According to a recent study, the GentleWave System was effective in retrieving separated instruments while conserving the dentinal structure [27].

The GentleWave system showed slightly cleaner canals among the treatment groups. However, the differences between the treatment groups were not statistically significant. Previous study by Wright et al. showed a similar result that GentleWave removed more residual obturation material than the side-vented needle and EndoVac, but the differences between GentleWave and the other two groups were not statistically significant [28]. The effectiveness of the PUI and GentleWave system in endodontic retreatment was recently reported [29]. The use of PUI and GentleWave significantly reduced the volume of remaining filling material after initial instrumentation. However, none of these techniques were able to render canals free from filling materials. When comparing the cleanliness of the middle and apical thirds by SEM analysis, there were no significant differences between ultrasonic instruments and GentleWave system, which is in agreement with the present study.

Results from this study should be interpreted with caution because of the large standard deviation observed. One possible explanation is the challenge of standardizing the amount of residual obturation material after conventional retreatment. In this study design of using extracted teeth, the difference in the anatomy of the root canal might have the influence on the differences in the amount of debris formed on the root canal surface. Moreover, the difference between groups may not be shown because of the low statistical power of this study induced by a small sample size and the large number of groups.

In addition, representative specimens in this study were sectioned horizontally and analyzed at 5 mm from the anatomic apex [30,31]. The results showed that the GentleWave System removed the sealer even from the dentinal tubules, while the presence of sealer at least 100 µm deep into the tubules was observed when the teeth were treated with an ultrasonic or sonic system. However, the penetration of the sealer after obturation was not always 100 µm into all tubules. This penetration into tubules may result from a cold lateral condensation technique and the primary cleaning of the root canals using conventional needle syringe irrigation [31]. Previously, the ability of the GentleWave System to penetrate approximately 450 µm into dentinal tubules was shown [32]. The results from our study support this penetration into the dentinal tubules. More studies are needed to confirm this finding.

Although SEM allows the highly detailed observation of dentinal tubules and filling material, a 3D view of the entire root canal system cannot be achieved by this method. Thus, this method did not allow the measurement of the thickness of either residue. Furthermore, SEM evaluations allow the assessment of only limited areas of the canal wall. Most of the studies assessed root canal filling materials removal efficiency by means of micro-computed tomography (micro-CT) [28,29,33,34,35]. Further investigation, which may include the use of other assessment techniques such as micro-CT, may provide a 3D analysis of the removal of obturation materials. Energy dispersive X-ray spectroscopy (EDX) may be used for the microchemical analysis of root canal surface, and thus it can be possible to identify the chemical elements included in the composition of gutta-percha and sealer debris [8].

This study presented the efficacy of the GentleWave System and the passive ultrasonic or sonic system in the removal of obturation debris. The GentleWave System showed higher cleaning efficacy of the apical and middle root canal walls compared to the control group but did not differ significantly with the tested passive ultrasonic or sonic irrigation method.

## Figures and Tables

**Table 1:** Mean debris and smear scores in the apical and middle regions and standard deviation (SD) of the six groups. Different superscript letters indicate statistically significant differences between groups (*p* < 0.05).

	Debris_Middle	Debris_Apical	Smear_Middle	Smear_Apical
Activation method	Mean (SD)	Mean (SD)	Mean (SD)	Mean (SD)
Group 1 (Negative control)	0.00(0.00) ^a^	0.32 (0.46) ^a^	1.50 (0.67) ^b^	2.82(0.93) ^b^
Group 2 (Positive control)	0.82(0.34) ^b^	1.14 (0.45) ^b^	3.45 (0.47) ^c^	3.55(0.52) ^b^
Group 3 (Piezon Master 700)	0.68(1.08) ^a,b^	0.82 (1.12) ^a,b^	1.09 (0.58) ^b^	1.41(0.80) ^a^
Group 4 (ENDOSONIC Blue)	0.23(0.41) ^a^	0.45 (0.69) ^a,b^	0.64 (0.60) ^a,b^	1.10(0.62) ^a^
Group 5 (EDDY)	0.45(0.42) ^a,b^	0.50 (0.45) ^a,b^	0.95 (0.91) ^a,b^	0.73(0.41) ^a^
Group 6 (GentleWave System)	0.14(0.32) ^a^	0.18 (0.40) ^a^	0.27 (0.41) ^a^	0.64(0.45) ^a^
